# The Technique of the Artificial Pneumothorax Treatment of Consumption

**Published:** 1913-03-29

**Authors:** 


### THE TECHNIQUE OF THE ARTIFICIAL PNEUMOTHORAX TREATMENT OF CONSUMPTION.

BY A SANATORIUM SUPERINTENDENT.

Despite its limited sphere, this treatment, on account of the unusualness of the basic plan, and because it does not necessarily posit early cases of the disease, often produces unmistakably favourable results in a short time, and perforce greatly modifies physical signs; and it has attracted very great notice on the Continent now that improvements in technique have minimised its dangers. The following methods are those demonstrated to the writer by Dr. Lillingston, at present the leading exponent in England of this treatment, and himself a notable example of a successful case.

The apparatus, supplied by Messrs. Allen and Hanbury, at three guineas, is very simple. A Wolff bottle A, a similar glass cylinder B, both rubber-corked, with rubber tubing connecting the long glass tube in each; B is doubly graduated (a needless refinement) so as to register both decrease in gas and increase in water content. The amount of that water is such that when A and B are on a level, and the tubing between full like a siphon, fluid reaches the 350-c.c. mark in B. All the upper part of B contains nitrogen gas. DD are glass tubes holding sterilised (in an ordinary oven) cotton-wool to act as a filter to the gas ere it enters the pleural space. All glass-rubber connections are bound round with thread to prevent leakage of wind or water. At E a glass T-piece is introduced to connect with the water manometer C. Fill this with water—coloured with red ink for plainness—till the fluid in each limb reaches O on the graduated scale. Then, as one faces the apparatus, rise of the left-hand red column, with corresponding fall of the right-hand one, show negative pressure; the reverse conditions, positive pressure. Beyond the second D filter one comes to what may be called the business end of the whole instrument. F is a glass plug closing, and therefore excluding sepsis from, the end of the rubber tube, with which, when all is ready, the needle connects. The rest of the illustration shows a portable wooden stand any carpenter can make for half-a-crown.

### Starting a New Case.

But, before all is ready for putting the needle into the chest, there are very many preliminaries: preliminaries directed to three great ends—avoidance of sepsis, avoidance of shock (technically called "pleural reflex"), and avoidance of a wrong destination for the nitrogen gas.

To begin with, let the patient have a quarter of a grain of morphine hypodermically. Then wash hands (no elaborate ritual) and dip them in antiseptic, and prepare two other hypodermics—the one of ether, as an improbably needed stimulant, the other of a third of a grain of eucaine in not more than 10 minims of sterilised water. While the latter is dissolving, re-auscult your patient and select three sites to go in at. Needless to say, the bad lung is the one for compression, so have that uppermost, and a couple of pillows under the axilla to arch laterally the thorax and open out the intercostal spaces. Generally speaking, one should keep clear of the most diseased part—the apex. Breath sounds should be audible, too, where the needle is to enter, and you must necessarily pick a spot over an intercostal space. Subject to these limitations, have the three spots as far apart as may be; paint them with tr. iodi to the size of pennies. While the iodine is drying wash hands again and inject the eucaine solution (small in volume so as not subsequently to cause escape of fluid into the lumen of the pneumothorax needle) progressively—that is, a little subcutaneously, a little further down, and most of it for the parietal pleura, penetration of which can often be recognised by a feeling as of puncture of a thin, toughish membrane. If it occurs, this is a good sign, as tending to show absence of adhesions. Lastly, inject a little just under the parietal pleura.

While the eucaine is acting, the next business is making dry the whole inside of the pneumothorax needle (Saugmann's). If moisture be present in the lumen, respiratory oscillations will not be communicated to the manometer, and we lose an indispensable guide. It is, therefore, best to keep the needles in absolute alcohol. Shake, put on an antiseptic cloth, and leave for a minute or two to let the spirit evaporate; otherwise a troublesome performance is necessary to expel all moisture. Then rub over with antiseptic the eighteen inches of tubing above F. Remove plug and put the tubing on to the angled opening of the needle; the straight opening is for the stilette, the length of which must now be tried in the needle, so as to refresh the memory with the exact length of the handle part left projecting above, when the thin end gets home in the hole at the needle-point. The shape of this, for first attempts, is the ordinary shape, the pattern of needle having an imperforate point and lateral hole, being used for refills. Pinch the tube near to F to see if the manometer responds briskly with oscillations.

Now touch the little hole left by the eucaine needle with iodine, stretch the skin, and go smartly in with a light, quick jab. Watch the manometer for any movement. When at the depth of an inch, use the stilette, opening and closing the little tap which admits it very quickly, to avoid inlet of unfiltered air. Give half a turn to the stilette to clear out anything present and withdraw, doing this after each advance. If the pleural space has been found, an oscillating negative pressure will show on the manometer, rather greater with inspiration, rather less on expiration. The great characteristic of pleural oscillations is that they are big ones, the mean being not less than 4 c.c. To make this last statement universally intelligible, it should be recalled that the scale depicted records pressures in cubic centimetres. A fall of, say, 5 c.c. in the left-hand limb means, however, a minus pressure of 10 c.c., because the right-hand column simultaneously rises 5 c.c. also. If −10 is the higher reading (with inspiration), then −8 may be the lower one, with expiration. So −10 and −8 will be the initial reading, which should be dictated to an assistant.

If one has gone too deep, and is in the lung, there may be respiratory oscillations—negative ones, too; but they will be much smaller—say, −2 and −1—the mean thus less than 4. An additional precaution is to put ol. menth. pip. in the water in the glass bottles. Then if, in spite of all precautions, nitrogen gas is sent into the lung, its peppermint flavour will be noticed by the patient at once. If the needle-point is in a vessel the manometer will show a positive pressure. The great thing is not to undo the pinchcock at X unless in presence of characteristic pleural oscillations; and hence the value of preparing three sites. If one fails with one, two others are available. If all three fail, try other three the next day.

When the stopcock is opened, and no kinks in the tubing occur, gas is sucked into the pleura. The oscillations at once become less. When 100 c.c. have gone in, pinch at X again, note a reading, and raise A so that its water-level is the same as that of B. Then open X (oscillations again diminish) and complete the initial dose, 250 c.c. Pinch, dictate the final reading (say, −7 and −4), withdraw needle, noting the depth to which it has entered, and touch the wound with iodine.

Make the record on case-sheet as follows:—

$$\left.\begin{matrix}-10 & -8\\ -7 & -4\end{matrix}\right\}250$$

### Refills.

With a typical case, refill on the next day. Obviously the first requisite is fresh ammunition—that is, one must put more nitrogen gas in B. To replenish B, clamp the length of rubber tubing between the two bottles. Turn on the nitrogen cylinder very slightly, take the rubber tubing off the end of the short glass tube of B, and fit on the tubing of the nitrogen cylinder. Quickly remove the clamp just mentioned, and watch the water sinking in B until it reaches the 350-c.c. mark. Turn off the nitrogen, disconnect, and quickly replace the tubing on the short glass tube of B aforesaid. Now the water in A and B is level, and the top part of B is full of nitrogen.

Omit the morphine, but again prepare eucaine, and examine while it is dissolving. Note extent of obliteration of breath sounds, character of percussion note, etc. Give the eucaine in the manner previously described, and at the very site of the successful injection. Again, while waiting for the eucaine to act, dry the needle; the pattern, however, having a lateral opening. As before, disinfect tubing, connect needle, try length of stilette, try sharpness of oscillations. Go in at the hole previously made. Proceed just as in a first injection, with dictation of first reading (note extent to which—perhaps to −9 −7—it has fallen from that attained yesterday), with "pinching" and taking intermediate readings, and with dictating a final reading (say, −5 and 0). 500 c.c. should be got in. So that the second note runs:—

$$\left.\begin{matrix}-9 & -7\\ -5 & 0\end{matrix}\right\}500$$

The second refill, on the next day, is the same, save that now we leave off the eucaine. 500 c.c. should be got in, and one should end up with +2 and +8. This moderate positive pressure, which will generally secure collapse of the lung, will be maintained by 400 c.c.—500 c.c. on the sixth day, thirteenth day, twentieth day, and thereafter every ten days, always being guided by the pressures observed. Be guided, too, by physical examination. The sharpest of eyes must be kept on the good lung, for *râles* have a way of appearing where they were never heard before. If this occurs, strict rest in bed and longer intervals and lower pressures are indicated. On the other hand, rapid lessening of the signs of pneumothorax, such as reappearance of breath sounds, is an indication for shortening the intervals; as is, too, quick absorption of gas, shown by rapid fall in pressure from one injection to another. Last, but by no means least trustworthy as a guide, comes the temperature record. If one has secured that rarest of therapeutic triumphs—namely, quick defervescence of long-continued pyrexial phthisis, and it is the distinction of artificial pneumothorax to do this—then, surely, as long as the temperature continues down, one should be inclined to play a waiting game, even if this entail stopping short of the orthodox +2 and +8. But this is a suggestion only. As to getting the patient up, proceed very gradually, beginning with half-an-hour sitting up in a dressing-gown, and that only after the temperature has been normal, in the strict sanatorium sense, for a week. Unlike another special form of treatment, of a much lower grade of efficacy, artificial pneumothorax is only recommended when combined with hygienic-dietetic measures.

### Adhesion Cases.

So far we have only considered the straightforward case; but there may be adhesions—total, obliterating the pleural space, or partial, forming pockets or cutting off apex or base. Total adhesion means failure to initiate treatment. With partial adhesions, as one would expect, the patient takes less gas, and the pressure quickly rises. In the intervals it quickly falls, too, for the gas being under pressure is more quickly absorbed. The limit to proceed to is +20 or thereabouts, certainly not more than +30. Intervals must be three or four days in place of a week or ten days, in order that a good positive pressure may be kept up in order to stretch the adhesions. Here again, however, signs of disturbance in the good lung may supervene and hinder this endeavour. High pressures, too, are especially likely to lead to effusion, and this brings up the subject of sequelæ.

Early sequelæ are cyanosis and breathlessness. Rise of temperature need not alarm, for the temperature is frequently higher the night of the first injection than before or afterwards. A good deal of breathlessness is natural enough, too. Later, if nausea and fever occur, examine for fluid, which comes on in a third of all cases. If half-way up the chest or less, leave it. If more, withdraw the fluid and replace with nitrogen, being guided by the ordinary indications, but having the patient sitting up, and inserting the aspirating needle and the nitrogen needle simultaneously, the latter high up, and the former low down. Temperature due to fluid, if the precautions detailed have been observed, soon falls; the fluid is never septic, and in adhesion cases, where, owing to the high pressures used, it is especially liable to occur, may even help in dilating the pleura.

MAXIMS AND GENERAL HINTS.

Never inject more than three days running, nor try more than four punctures at a sitting, for fear of pleural reflex.

When in doubt where the point of the needle is, make plenty of use of the stilette, even as a probe, to feel the parietal pleura; always do this before deciding to go deeper.

One usually reaches the pleura when a little less than half the needle-shank is left projecting. Thick chest walls are naturally not often encountered.

Pinch and take a reading every 100 c.c. run in, or oftener in adhesion cases. Always clamp before raising A. With positive pressures A may have to be high above B; a retort stand to fit A is then useful.

Points to make one suspicious that gas is entering lung instead of pleura are, besides those already mentioned, only getting in a small amount. 500 c.c. run in easily with slowly rising pressure excludes lung entry of gas. If violent coughing occur, or any *contretemps*, whip the needle out.

When putting the apparatus away, leave with the water in A and B level, otherwise gas leaks are invited. Keep the cylinder in its cocoanut matting; if dropped when uncovered it may burst.

It will last a year doing three or four cases; when away being recharged, atmospheric air answers well to use; the rate of absorption is not much more rapid.

